# Effectiveness of a novel traction device in endoscopic submucosal dissection for colorectal lesions

**DOI:** 10.1007/s00464-022-09228-4

**Published:** 2022-08-08

**Authors:** Xiao Liu, Xinying Yu, Yanbin Wang, Jianfeng Yu, Xinjuan Liu, Zhen Liu, Jianyu Hao

**Affiliations:** 1 Department of Gastroenterology, Beijing Chaoyang Hospital, Capital Medical University, 8 Workers Stadium South Road, Chaoyang District, Beijing, 100020 People’s Republic of China; 2 Department of Gastroenterology, Beijing Tiantan Hospital, Capital Medical University, No. 119 South Fourth Ring West Road, Fengtai District, Beijing, 100070 People’s Republic of China

**Keywords:** Endoscopic submucosal dissection (ESD), Traction device, The total procedure time (TPT), Submucosal dissection time (SDT), Submucosal dissection speed (SDS)

## Abstract

**Background:**

Among all types of superficial gastrointestinal (GI) neoplasms, colorectal lesions are recognized as one of the most difficult locations to operate, due to the limited operation space, physiological bends, poor visualization of the submucosal dissection plane sheltered by colorectal crinkle wall, and the thin intestinal mucosa layer which is easy to perforation. The purpose of this prospective study is to evaluate the feasibility, efficacy, and safety of a novel endoscopic traction technique in assisting the endoscopic submucosal dissection (ESD) procedure in colorectal lesions.

**Method:**

A total of 117 patients with colonic lesions who underwent endoscopic treatment were enrolled between August 2020 and January 2021 at the endoscopic center of Beijing Chao-yang Hospital of Capital Medical University. Based on whether traction device was used during the operation, 60 and 57 patients were assigned to the conventional ESD group and clips and rubber band triangle traction-assisted ESD group (CRT-ESD, in which three clips and a rubber band were used to form an elastic triangular traction device), respectively. The total procedure time (TPT), submucosal dissection time (SDT), submucosal dissection speed (SDS), and rate of adverse events of the two groups were analyzed.

**Results:**

After excluding patients who did not undergo treatment (conventional ESD, 1; CRT-ESD, 4), 112 patients were included in the study (conventional ESD, 59; CRT-ESD, 53). The baseline characteristics of the patients were well balanced between the two groups. The TPT (58.71 ± 26.22 min vs 33.58 ± 9.88 min, *p* < 0.001) and SDT (49.24 ± 23.75 min vs 26.34 ± 8.75 min, *p* < 0.001) were significantly different between the conventional ESD group and CRT-ESD group. The CRT-ESD group had significantly higher SDS than that of the traditional ESD group (0.54 ± 0.42 cm^2^/min vs 0.89 ± 0.40 cm^2^/min, *p* < 0.001). There were 4 (6.8%) cases of perforation in the traditional ESD group, and no perforation occurred in traction-assisted ESD.

**Conclusions:**

Compared with traditional ESD, CRT-ESD with clip and rubber band is both safer and more effective in the treatment of colorectal lesions.

**Graphical abstract:**

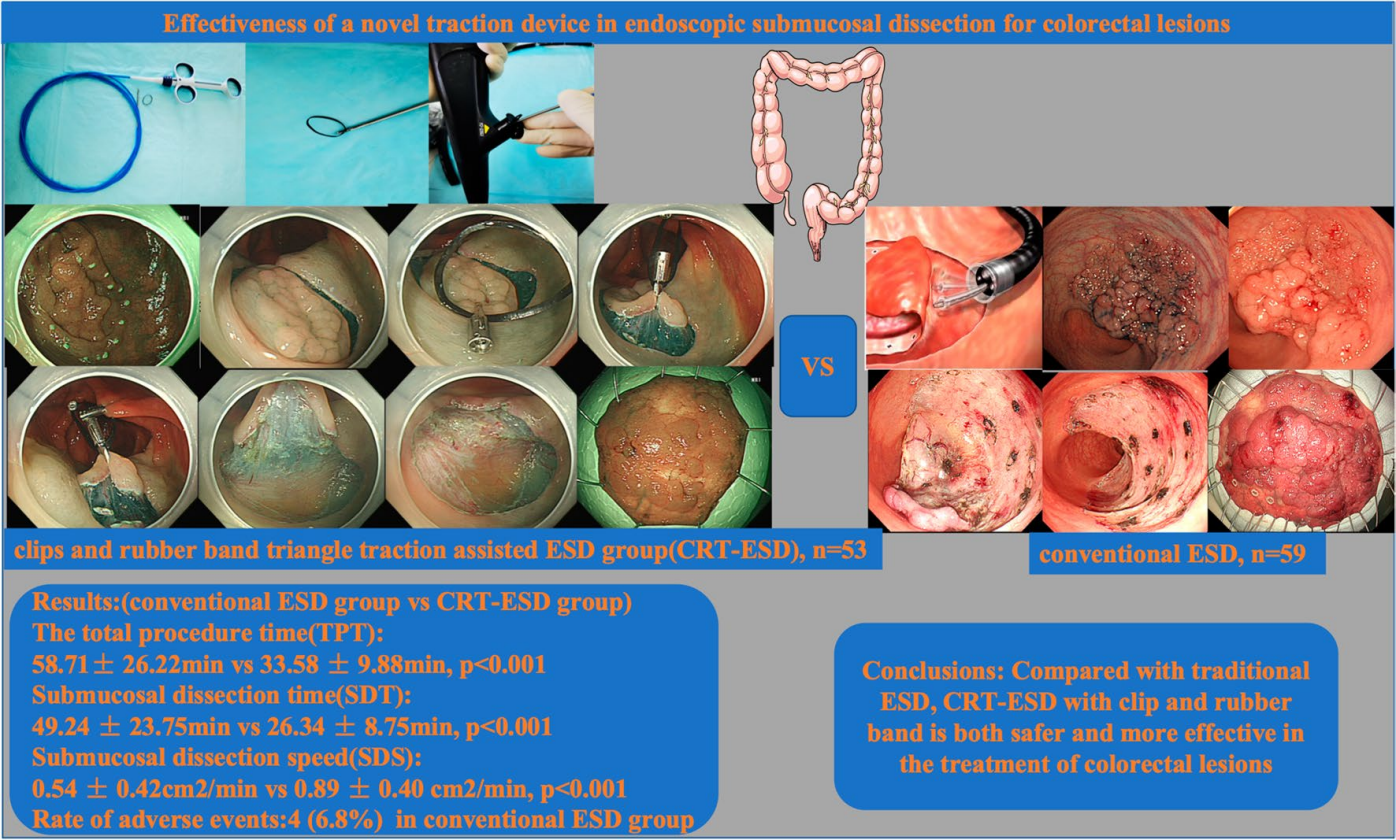

Endoscopic submucosal dissection (ESD) represents an advanced therapeutic technique aiming to provide minimal invasiveness and favorable outcomes in patients with superficial GI neoplasms [1–5]. However, the application of ESD in clinical practice is still limited by the complexity in operation and the long time of the cultivation [6]. In addition to the correct recognition of the mucosal and submucosal layers, tumor location has been reported as the most significant factor associated with prolonged operation time for ESD. At present, many factors contribute to the recognition that colorectal lesions are the most difficult location of superficial GI neoplasms. These factors include limited operation space, multiple physiological bends, poor visualization of the submucosal dissection plane sheltered by colorectal crinkle wall, and the thin intestinal mucosa layer which is easy to perforation [7].

Methods for creating traction have been developed to overcome the challenge [8–11]. Traction can effectively pull the mucosal layer apart, which leads to faster visualization of the submucosal dissection plane, also called “the third hand of endoscopic treatment”. With these characteristics, traction could speed up ESD operation and reduce the probability of perforation. Several techniques of traction have been tested and reported, such as clip with line method [8], spring-and-loop with clip (S–O clip) method [11], clip and snare method [12], and magnetic method [13–15]. These methods facilitate dissection, but they either require additional maneuvers such as reinserting the endoscope, or expensive adjunct devices that are not available in most hospitals.

We improved the previous traction methods and developed a new firm triangle traction device using clips and a rubber band. The traction device has some advantages. First, the materials of this traction device are affordable and accessible. Second, the device is easy to create and could be used without reinserting the endoscope. It also maintains good tension and stability throughout the whole procedure with the triangle structure. In this case series study, we aim to evaluate whether the clips and rubber band triangle traction-assisted ESD (CRT-ESD) improves clinical outcomes in treating colorectal lesions, compared with the conventional ESD.

## Materials and methods

### Patients

All ESDs were planned according to the Japanese guidelines for ESD of colorectal cancer [16] by the same endoscopist. All patients signed informed consent voluntarily. The absolute indications for endoscopic treatment are colorectal adenoma and intramucosal carcinoma without lymph node or vascular metastasis. The relative indication is SM1 carcinoma with slight invasion to the submucosa (the depth of submucosal invasion ≤ 1000 μm). All lesions were evaluated by endoscopic ultrasonography (EUS) and magnifying endoscopy (ME) before the operation to ensure that the depth of invasion was within the indications. We recommended that ESD should be used in the treatment of lesions with a maximum diameter larger than 20 mm, which must be resected once under endoscopy. Exclusion criteria were as follows: impossible cessation of anticoagulant or antiplatelet medications; active infection; pregnancy or breastfeeding; severe mental disorder; unstable hypertension, heart disease or uncontrollable diabetes mellitus.

All participants provided written informed consent before enrollment. The study has been approved by the Ethics Committee of Beijing Chao-yang Hospital. The clinical trial has been registered at the Chinese Clinical Trial Registry with registration number ChiCTR2000040258.

### Randomization and data collection

The sample size of this study was calculated based on the primary point of submucosal dissection speed. A pre-test (10 cases in both groups) was carried out to evaluate the expected speed of the two ESD methods. In our pre-study, the speed for conventional ESD and CRT-ESD was 0.35 ± 0.17 and 0.26 ± 0.04, respectively. Patients were assigned to receive either conventional ESD or CRT -ESD at a 1:1 allocation ratio. Based on a two-sample t-test, a sample size of 94 subjects treated with conventional ESD or CRT-ESD was calculated as necessary to ensure more than 90% power for a 2-sided significance level of 5%.

### Clips and rubber band triangle (CRT) traction device design

**Fig. 1:**
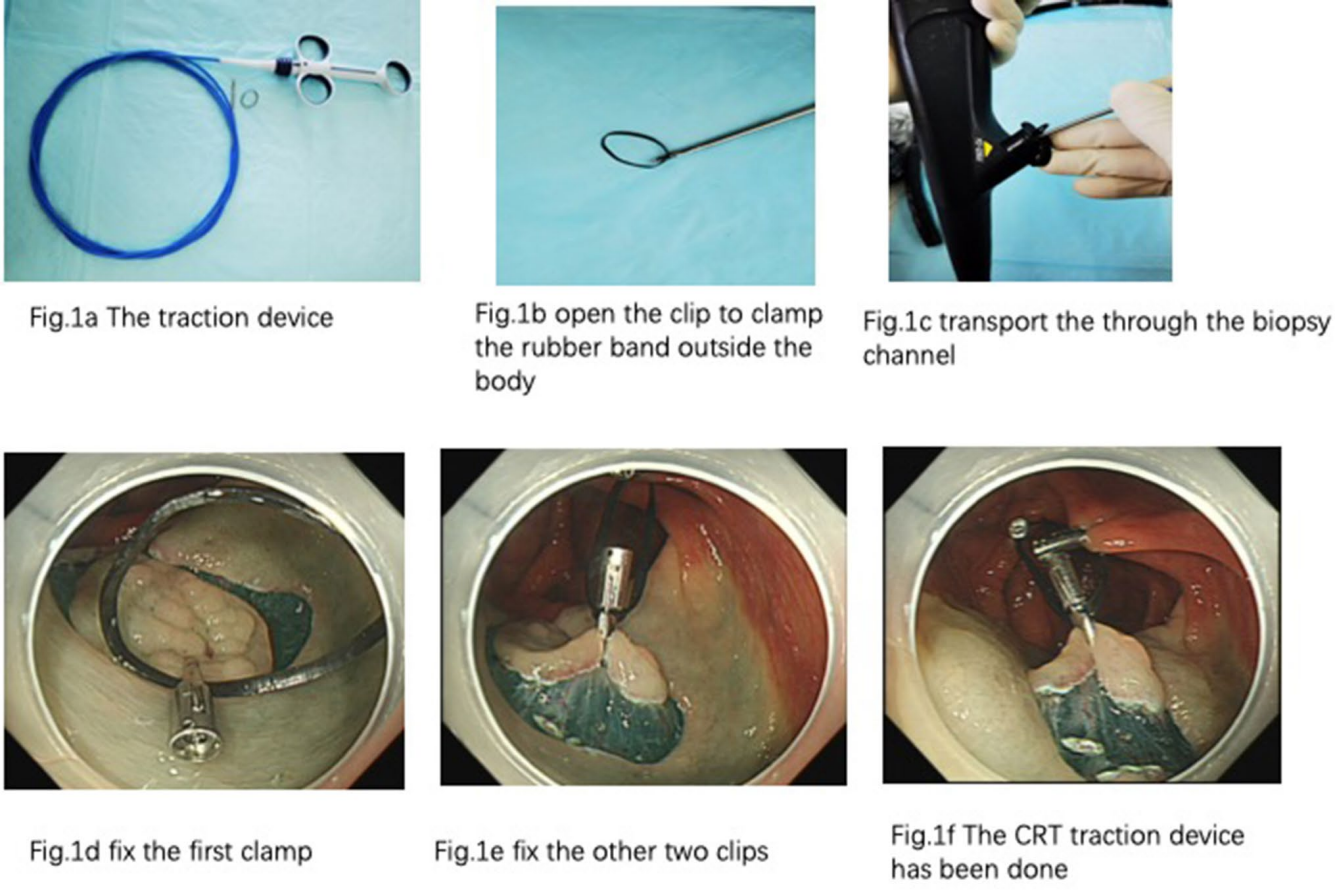
The consists of the traction device outside and inside the body

The traction device consists of three clips and a rubber band (Fig. 1a). The clips are the harmonious clips of Nanwei Medical Co., Ltd (medium-sized, opening diameter ≥ 10 mm). The rubber band is customized by the manufacturer to be of 2 cm in diameter and 3 mm in thickness, which allows it to pass through the biopsy channel of endoscope.

**Fig. 2:**
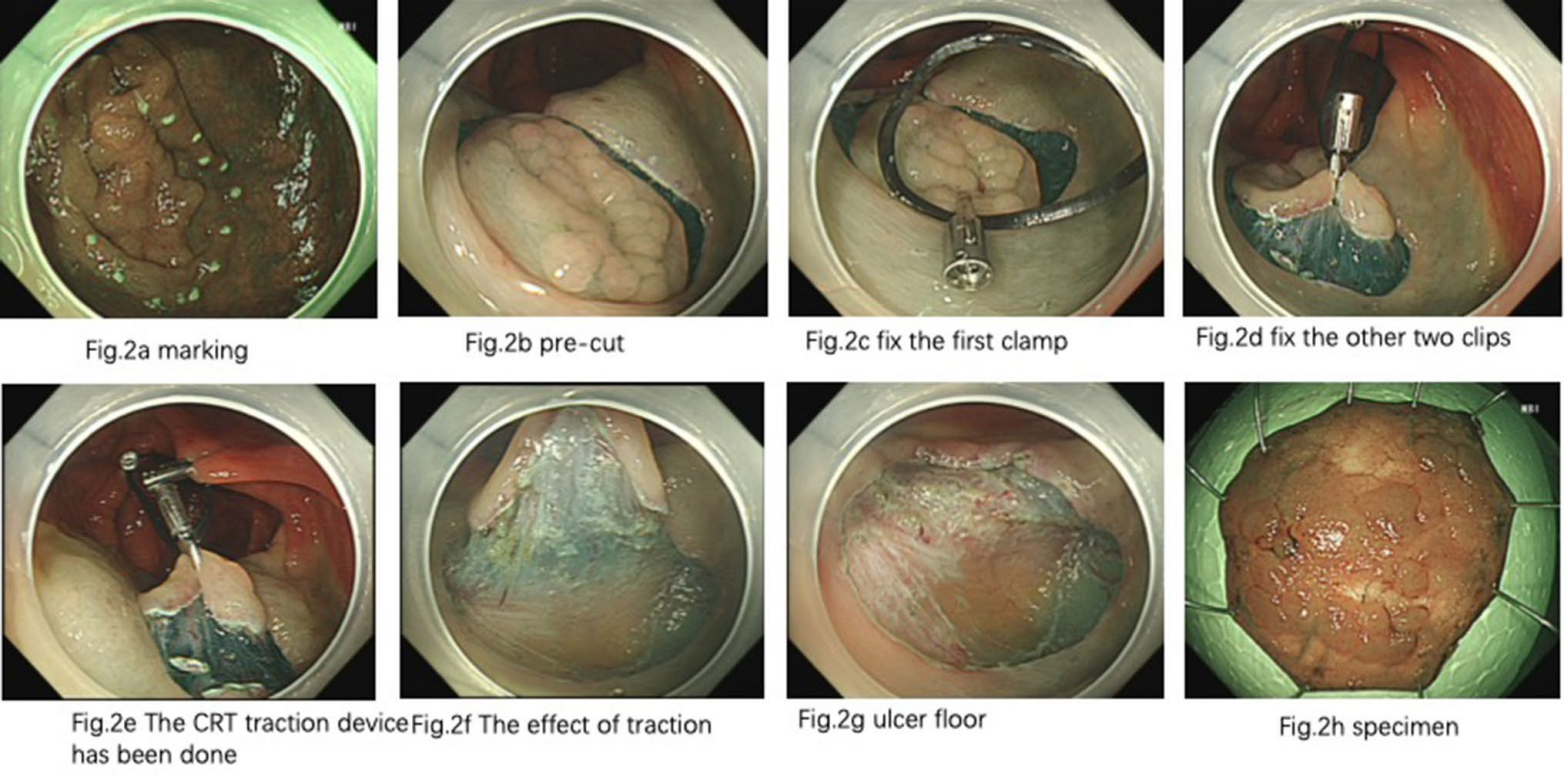
CRT-ESD procedures

The procedure to build the CRT traction device: (1) Open the clip to clamp the rubber band outside the body(Fig. 1b); (2) Transport it to the operation area through the biopsy channel (Fig. 1c); (3) After the rubber band position is preliminarily determined by the operator, the clip holding the rubber band is clamped onto the pre-cut mucosa, and the first clamp is fixed (Fig. 1d); (4) Depending on the need of the operation, the other two clips are fixed at other positions of the intestinal wall (Fig. 1e). The final CRT traction device is shown in Fig. 1f.

### Conventional ESD and CRT -ESD procedures

All ESD procedures were performed by one experienced endoscopist with a single-channel endoscope (Olympus). Conventional ESD techniques have been described in detail in many articles [17, 18]. The conventional procedures of ESD included: (1) a transparent cap was fixed in the front of an endoscope; (2) circumferential markings were made at least 5 mm from the lateral side of the tumor margin with a dual knife (KD-650U; Olympus); (3)normal saline mixed with indigo carmine(0.4%) and diluted epinephrine (1:100,000,0.01 mg/ml) was injected into the lesion by using a Boston entry needle(25G); (4) Cutting was done along the outside the markers with a dual knife (KD-650U; Olympus) until the submucosal layer was removed; (5) all visible vessels on the ulcer floor were coagulated with a coagrasper (Olympus FD-410LR).

During the CRT-ESD procedures, marking, injection and the pre-cut process are the same as that of conventional ESD. After the pre-cut process, the CRT traction device was fixed in the operation area according to the design mentioned above. The release position of the traction device needs to be decided by the operator according to the needs of the operation. The release process has undergone simple training. After the dissection process and the ulcer floor were coagulated, the clips on the intestinal wall were removed with foreign forceps (Fig. 2). The specimen was sent for pathological analysis by an experienced pathologist independently of the endoscopists.

### Evaluations and definitions

Patients’ age, sex, tumor’ location as well as the endoscopic type were collected as baseline information. Other baseline characteristics include the specimen length, specimen width, spacemen size, and differentiated degree.

ESD total procedure time (TPT) was defined as the time from the start of submucosal injection to the end of tumor removal. In order to compare the dissection time of the two groups accurately. The submucosal dissection time (SDT) for the CRT-ESD method was defined a as the time from the end of the traction device’s placement until the completion of tumor dissection. Conventional ESD’s SDT was from the end of the mucosal pre-cut to completion of tumor dissection. The specimen area was calculated by multiplying the major axis radius, minor axis radius and π, the major axis radius and the minor axis radius were measured from the postoperative specimen. We calculated submucosal dissection speed (SDS) by dividing the area of the resected specimen by SDT [7].

En-bloc resection was defined as the removal of the whole tumor in a single piece. R0 resection was defined as en-bloc resection with lateral and vertical margins free of tumor cells. The curative resection was defined as R0 resection without risk factors, which included non-lymphaticvascular invasion and the depth of submucosal invasion ≤ 1000 μm [19].

We defined adverse events as perforation, delayed bleeding, abdominal pain and fever. Perforation was defined as full-thickness defects of the colorectal wall, which were recognized by the operator as a state in which connective tissue, adipose tissue, and/or serosa were visible through the defect. Perforation was divided into intraoperative perforation and delayed perforation. Intraoperative perforation refers to the perforation during ESD, and delayed perforation refers to the perforation after ESD. Delayed bleeding was defined as the presence of marked bloody stool after treatment or the requirement for hemostasis after surgery [20]. We use the digital pain scoring method. The numbers 0–10 represent the degree of pain, in which 0 represents no pain, 1–3 represents mild abdominal pain (does not affect sleep), 4–6 represents moderate abdominal pain, 7–9 represents severe abdominal pain (unable to sleep or wake up from sleep), and 10 represents severe pain. Body temperature was measured by axillary temperature, of which 37.3–38 °C were low fever, 38.1–39 °C were moderate fever, and 39.1–41 °C were high fever.

Clip slip-off was counted when the clip came off from the lesion before the end of the procedure. Traction-related damage to the specimen was damage caused by the CTR traction device or the intestinal mucosa was damaged when the clip was removed.

### Histopathological evaluation

ESD specimens were stretched, pinned onto a cystosepiment, and totally immersed in 10% neutral buffered formalin for fixation. To assess both lateral and vertical margins, the specimen was cut into slices every 2 mm, parallel to a line that included the closest resection margin of the specimen. Exact carcinoma size, degree of differentiation, depth of tumor infiltration and presence of lymphovascular invasion were evaluated according to the Japanese Society for Cancer of the Colon and Rectum (JSCCR) Guidelines [20].

### Statistical analysis

Shapiro–Wilk test was used to assess the normal distribution of continuous data. Continuous data are presented as the mean ± standard deviation or median and interquartile range, as appropriate. Categorical variables are presented as numbers and percentages. Statistical significance was calculated by the Student T test or Mann–Whitney U test for continuous data. Pearson chi-squared test or Fisher exact test were used for categorical data. All analyses used SPSS software, version 25 (IBM Inc., New York, NY, USA).

## Results

### Patient characteristics

A total of 117 patients were enrolled between August 2020 and January 2021 at the endoscopic center of Beijing Chao-yang Hospital, Capital Medical University. They were randomly divided into two groups using the random numbers generated in excel, 60 and 57 patients were divided into the conventional ESD group and CRT-ESD groups respectively. After excluding patients who did not undergo treatment (conventional ESD, 1; CRT-ESD, 4), 112 patients were included in the analysis.

The baseline characteristics of the patients were listed in Table 1. The mean age and male proportion had no statistically difference. Notably, there were no significant differences between the two groups in terms of tumor location and endoscopic type. The average specimen size was 28.49 ± 20.04 cm^2^ and 29.28 ± 15.56 cm^2^ in the conventional ESD and CRT-ESD groups, respectively. The specimen length, specimen width, specimen size and the differentiation degree were well balanced between the two groups.

**Table 1:** Baseline characteristics

Characteristics	Conventional ESD (*n* = 59)	RCT-ESD (*n* = 53)	*p* value
Age, years	67.37 ± 9.15	64.49 ± 11.93	0.152
Sex, male	26 (44.1)	30 (56.6)	0.256
Tumor location			0.249
Rectum	13 (22.0)	17 (32.1)	
Sigmoid colon	10 (16.9)	12 (22.6)	
Descending colon	12 (20.3)	6 (11.3)	
Transverse colon	9 (15.3)	3 (5.7)	
Ascending colon	7 (11.9)	10 (18.9)	
Ileocecal part	8 (13.6)	5 (9.4)	
Endoscopic type			0.383
Elevated (0-I, 0-IIa)	31 (52.5)	26 (49.1)	
Depressed (0-IIb, 0-IIc, 0-III)	3 (5.1)	7 (13.2)	
Mixed (0-lla+llc, 0-llc+lla)	1 (1.7)	0 (0)	
LST	24 (40.7)	20 (37.7)	
Specimen length, cm	3.24 ± 1.22	3.29 ± 1.10	0.590
Specimen width, cm	2.53 ± 0.95	2.69 ± 0.65	0.068
Specimen size, cm^2^	28.49 ± 20.04	29.28 ± 15.56	0.177
Differentiation degree			0.850
Low grade intraepithelial neoplasia	27 (45.8)	22 (41.5)	
High grade intraepithelial neoplasia	21 (35.6)	22 (41.5)	
Adenocarcinoma	11 (18.6)	9 (17.0)	

### Outcomes of conventional ESD and CRT-ESD

Clinical Outcomes of conventional ESD and CRT-ESD were shown in Table 2. Significantly differences were observed between conventional ESD and CRT-ESD in terms of total procedure time (58.71 ± 26.22 min vs 33.58 ± 9.88 min, *p* < 0.001) and submucosal dissection time (49.24 ± 23.75 min vs 26.34 ± 8.75 min, *p* < 0.001). As to the submucosal dissection speed, the CRT-ESD group was faster than the conventional ESD group (0.54 ± 0.42 cm^2^/min vs 0.89 ± 0.40 cm^2^/min, *p* < 0.001).

**Table 2:** Clinical Outcomes of conventional ESD and CRT-ESD

Outcome	Conventional ESD (*n* = 59)	RCT-ESD (*n* = 53)	*p* value
Total procedure time, min	58.71 ± 26.22	33.58 ± 9.88	< 0.001
Submucosal dissection time, min	49.24 ± 23.75	26.34 ± 8.75	< 0.001
Submucosal dissection speed, cm^2^/min	0.21 ± 0.17	0.37 ± 0.18	< 0.001
En-bloc resection	58 (98.3)	53 (100.0)	1.000
Curative resection	50 (84.7)	50 (94.3)	0.131
R0 resection			
Horizontal margin involvement	2 (3.4)	2 (3.8)	0.649
Vertical margin involvement	3 (5.1)	1 (1.9)	0.351
Histologic depth of tumor			0.389
Mucosa	49 (83.1)	46 (86.8)	
Submucosa	10 (16.9)	7 (13.2)	
Adverse events			
Intraoperative Perforation	4 (6.8)	0 (0)	0.120
Delayed Bleeding	2 (3.4)	0 (0)	0.497
Abdominal pain	4(4–5)	4(3–4)	0.000
Fever	37.3 (37.1–37.5)	37.3 (37.1–37.5)	0.824
Clip slip-off during RCT-ESD	NA	3 (5.7)	NA
Traction-related damage to specimen	NA	1 (1.9)	NA

In the CRT-ESD group, clip slip-off and traction-related damage to the specimen were observed in 3 (5.7%) and 1 (1.9%) of 53 patients, respectively. The reasons for clip slip-off were excessive traction, and the traction-related damage was due to mucosal damage caused by foreign forceps when pulling off the clip. Mucosal injury was clamped with clips. There were four intraoperative perforation (6.8%) in the conventional ESD, perforation occurred because the lesions were located in physiological bends and the surgical area was not exposed well after repeated attempts, and there was no delayed perforation after ESD in both groups. There were two delayed bleeding (3.4%) in the conventional ESD. The abdominal pain score of traditional ESD group vs CRT-ESD group was 4 (4–5) vs 4 (3–4), *p* = 0.000, and the results were statistically significant. However, the body temperature of traditional ESD group vs CRT-ESD group was 37.3 (37.1–37.5) vs 37.3 (37.1–37.5), *p* = 0.824, and the results were not statistically significant.

All patients in the CRT-ESD group had undergone en-bloc resection, and one patient in the conventional group was cut off with two pieces. Because the lesion was located in physiological bend, the submucosal dissection plane cannot be exposed clearly, which led to the transportation from ESD to EMR-CAP.

There were 5 patients with margin involvement in the conventional ESD group (2 horizontal margin involvement and 3 vertical margin involvement) and 3 patients in the CRT-ESD group (2 horizontal margin involvement and 1 vertical margin involvement). For the 4 patients having vertical margin involvement with submucosal invasion depth ≥ 1000 μm, all of them underwent surgery treatment, and no residual cancer nor regional lymph node metastasis. The horizontal margin could not be judged clearly because of electrotome cauterization. These 4 horizontal margin involvement patients were followed up for more than half a year, and no tumor recurrence nor lymph node metastasis occurred. No significant differences between conventional ESD and CRT-ESD were seen in the histologic depth of tumor.

## Discussion

ESD helps patients with large superficial colorectal neoplasms to avoid surgery. However, due to the limited operation space, physiological bends, poor visualization of the submucosal dissection plane sheltered by colorectal crinkle wall, and the thin intestinal mucosa layer which is easy to perforation, colorectal lesions are recognized as the most difficult location of superficial GI neoplasms. The purpose of this prospective study is to evaluate the feasibility, efficacy, and safety of a novel endoscopic traction technique in assisting ESD procedure in colorectal lesions.

Several techniques of traction have been reported, such as clip with line method [8], clip and snare method [12], and magnetic method [13–15], and spring-and-loop with clip (S–O clip) method [11]. Although these traction methods can assist the operation process to some degree, clip with line method, clip and snare method and S–O clip) method require a reinsertion of the endoscope. Additionally, traction assistance is often effective at the beginning of ESD procedure, but the traction tension gradually reduces during the operation. Magnetic traction is a new method of traction which does not need a reinsertion of the endoscope and the traction tension will always effective during the ESD procedure. However, magnetic traction is not available in most institutions due to the high price.

Based on the improvement of various traction methods previously reported, we have created a novel traction method in this study which called CRT traction. The CRT traction device consists of three clips and a rubber band. Firstly, for proximal colon lesions, intraluminal traction has more auxiliary effect than extraluminal traction [21]. Secondly, some traction devices that have been reported require a reinsertion of the endoscope, resulting in prolonged operation time and even intestinal spasm, which makes the operation more difficult [8]. The customized rubber band we used in our study can pass through the endoscopic biopsy channel, which can save a lot of operation time without increasing the risk of intestinal spasms for proximal colon lesions. As we all know, a triangle shape is the most stable structure in terms of geometry. The traction device used in this study is a triangular structure composed of clips and rubber bands according to the theory of Physics. Stable traction devices not only have better traction efficiency but also can guide the direction of resection, which is very helpful for learners. Most important, most of the reported traction devices using dental floss or snare only have traction tension at the beginning of resection, and the traction force will gradually reduce as the specimen is stripped [12]. The rubber band used in our study has elasticity and can maintain traction tension throughout the entire operation process. Some experts have raised the question that the rubber’s elasticity will also decrease with the resection going on, and whether the reduction of elasticity will lead to a decrease in traction efficiency. In practice, we found that because of the thin intestinal mucosa, a slight tractive power can maintain an efficient traction. So even if the elasticity of the rubber band decreases with the resection of the lesion, it can still play a traction role throughout the entire operation process.

Several techniques of traction have been reported using SDS as the main evaluating indicator to analyze the effectiveness, finding that the SDS by ring-thread traction method, S–O clip, and clip-and-thread method were 23.5 mm^2^/min, 23.0 mm^2^/min, and about 29.0 mm^2^/min, respectively [22, 23]. In this study, we calculated the SDS by using their procedure time and the size of colorectal lesions, and demonstrated that the TPT was 58.71 ± 26.22 min, the SDT time was 49.24 ± 23.75 and the SDS was 0.54 ± 0.42 cm^2^/min in conventional ESD group, which was in the least inferior to CRT-ESD group. However, we can see a great improvement in endoscopic performance in the CRT-ESD group in terms of the TPT, SDT and SDS, which was 33.58 ± 9.88 min, 26.34 ± 8.75 min, and 0.89 ± 0.40 cm^2^/min respectively. We conclude that the CRT-ESD traction method is not only significantly better than the conventional ESD group, but also faster than the traction methods that have been reported in terms of SDS.

One patient in the conventional ESD group was cut off with two pieces which had not undergone en-bloc resection. Because the lesion was located in physiological bend and the submucosal dissection plane cannot be exposed clearly, so we change ESD to EMR-CAP for safety. The en-bloc resection rate in the CRT-ESD group was 100%. We can predict that proper traction can ensure the en-bloc resection of the difficult position of colorectal lesion. This is very important for objective histological evaluation.

Perforation during ESD will prolong the procedure time for endoscopic closure and increase the difficulty of operation. Furthermore, it causes peritonitis after ESD, requiring salvage surgery to prevent fatal clinical outcomes. Delayed bleeding can also prolong the hospitalization time and increase the pain of patients. In this study CRT-ESD group showed that no patient had perforation or bleeding, which is not only lower than that conventional ESD group (6.8%) in our study but also lower than those noted in previous studies(incidence of perforation and bleeding was 4.5% and 2.7%, respectively) [24]. This is not only because the traction device we used in this study has the normal effect as previously reported, but also the rubber band has efficient traction throughout the whole operation process. Most importantly, the physically stable triangular structure has better traction efficiency, and can guide the direction of resection, which has never been reported before. Though there were 3 clip slip-off and 1 traction-related damage, none of them had serious consequences. Therefore, we do not consider them as negative effect. This particular advantage of CRT-ESD may help to promote the adoption of ESD in general clinical practice.

There was no delayed perforation in both groups, so there was no statistical significance in postoperative body temperature between the two groups. The postoperative abdominal pain scores of the two groups were within the tolerable range, but compared with the CRT-ESD group, the abdominal pain in the traditional ESD group was more obvious. This is mainly related to the longer operation time of traditional ESD, which leads to more inflation and more stimulation to the intestine. Therefore, the use of traction can effectively reduce the operation time and reduce the symptoms of postoperative abdominal pain.

This study has some limitations. Firstly, this study was conducted in a single medical center without being widely verified by peer facilities. Therefore, the multicenter study will be required to confirm the effectiveness of CRT traction method. Secondly, the sample size of this study is relatively small, many indicators do not reflect statistical significance, such as perforation and bleeding rate, and large sample size research is needed to provide more objective indicators in the future.

In summary, CRT traction device is an effective tool in improving TPT, SDT and SDS. To some degree, this traction method can reduce the incidence of perforation, bleeding and other adverse events. Further randomized control trial in more facilities and expand sample size studies is necessary.
